# Research hotspots and frontiers of glymphatic system and Alzheimer’s disease: a bibliometrics analysis

**DOI:** 10.3389/fnagi.2025.1579373

**Published:** 2025-07-02

**Authors:** Shichao Liu, Lvping Zhuang, Xiaochun Li

**Affiliations:** ^1^Department of Neurosurgery, Fujian Medical University Union Hospital, Fuzhou, China; ^2^Fujian Key Laboratory of Molecular Neurology and Institute of Neuroscience, Fujian Medical University, Fuzhou, China; ^3^School of Basic Medical Sciences, Fujian Medical University, Fuzhou, China

**Keywords:** Alzheimer’s disease, glymphatic system, bibliometrics, CiteSpace, research trends

## Abstract

**Background:**

As a predominant neurodegenerative disorder, Alzheimer’s disease (AD) has garnered increasing attention regarding the association between its pathological mechanisms and glymphatic system (GS) dysfunction.

**Objective:**

This study employs bibliometric methods to systematically analyze the evolutionary trajectory and emerging frontiers of GS-AD research from 2010 to 2025, aiming to provide insights for clinical applications and scientific research.

**Methods:**

A total of 595 articles were selected from the Web of Science Core Collection. Knowledge mapping was constructed using tools such as CiteSpace and RStudio to analyze country/institutional collaboration networks, co-cited references, and keyword clustering.

**Results:**

Over the past decade, publication output in this field has demonstrated exponential growth. The United States maintains academic dominance, with the University of Rochester and the Nedergaard team serving as central research forces. While China ranks second in publication volume, its international influence requires further enhancement. High-frequency keyword analysis revealed three major research directions: anatomical mechanisms of the GS, (cerebrospinal fluid-interstitial fluid exchange, Aquaporin-4 polarization regulation), pathophysiological associations (amyloid-β/tau clearance, sleep-aging interactions), and clinical translational potential (diffusion tensor image analysis along the perivascular space (DTI-ALPS) imaging biomarkers, targeted intervention strategies). Co-citation analysis indicated that foundational studies by Iliff Jeffrey J and Nedergaard Maiken continue to guide the field, while recent research hotspots concentrate on glymphatic function assessment technologies and cross-disease mechanistic investigations.

**Conclusion:**

GS research is transitioning from fundamental mechanisms to clinical diagnostics and therapeutics. Future advancements necessitate the integration of multimodal imaging technologies and interdisciplinary collaboration to facilitate early AD diagnosis and therapeutic target development. This study represents the first systematic construction of a knowledge framework for this field, providing theoretical foundations for optimizing research strategies and translational pathways.

## Introduction

Alzheimer’s disease (AD), the most prevalent neurodegenerative disorder, currently affects over 50 million individuals worldwide and ranks as the sixth leading cause of death, posing a significant public health challenge (Ramachandran et al., 2021; Alzheimer’s Association, 2024). Its hallmark neuropathological features include extracellular amyloid-β (Aβ) plaques and intracellular neurofibrillary tangles mediated by hyperphosphorylated tau protein (Selkoe, 2003). Despite three decades of basic research that have profoundly advanced our understanding of AD pathogenesis, debates persist regarding its core molecular pathways and therapeutic targets. Recent clinical trials of immunotherapies using monoclonal antibodies—lecanemab [Leqembi™], donanemab (Sims et al., 2023; Van Dyck et al., 2023) and aducanumab [Aduhelm™](Budd Haeberlein et al., 2022)—demonstrate that clearing cerebral Aβ aggregates can slow early AD progression. These findings provide the first clinical-pathological evidence supporting disease-modifying therapy for AD. However, their limited clinical benefits may stem from delayed intervention or insufficient antibody dosage to achieve Aβ clearance thresholds required for meaningful outcomes (Jucker and Walker, 2023).

Notably, emerging research highlights the critical role of impaired glymphatic clearance of neurotoxic metabolites and misfolded proteins in AD pathogenesis (Nedergaard and Goldman, 2020). The glymphatic system (GS), a unique fluid transport network, facilitates metabolic waste clearance via perivascular spaces (PVS) formed by astrocytic endfeet (Simard et al., 2003). These PVS channels connect to the subarachnoid space, enabling deep brain access (Mestre et al., 2018b). Functionally analogous to peripheral lymphatics, this astrocyte-regulated cerebrospinal fluid (CSF)-interstitial fluid (ISF) exchange mechanism was first elucidated in rodent models by Iliff et al. (2012)(Iliff et al., 2012). Its efficiency depends on Aquaporin-4 (AQP4) water channel polarization at astrocytic endfeet, governing the directional CSF-ISF flow that removes neurotoxic species like Aβ oligomers and phosphorylated tau (Mestre et al., 2018a). Recent advances reveal that glymphatic dysfunction not only exacerbates Aβ and tau-mediated synaptic toxicity (Xie et al., 2013) but also bidirectionally interacts with sleep–wake cycles, aging, and cerebral hemodynamics (Hablitz et al., 2020). Animal studies demonstrate that AQP4 gene knockout or aging-induced glymphatic dysfunction significantly accelerates Aβ pathological deposition and induces hippocampal-dependent spatial memory deficits (Kress et al., 2014). Clinical imaging using diffusion tensor image analysis along the diffusion tensor image analysis along the perivascular space (DTI-ALPS) confirms reduced glymphatic activity in AD patients, correlating with decreased CSF Aβlevels and lower cognitive scores (Taoka et al., 2017; Chang et al., 2023). Collectively, these multidimensional findings underscore the GS’s pivotal role in AD’s pathological cascade, suggesting that targeting cerebral clearance mechanisms may offer novel therapeutic strategies.

Against the backdrop of accelerating global population aging, the GS, a pivotal discovery in neuroscience in recent years, has unveiled novel perspectives for developing innovative diagnostic and therapeutic strategies through its potential association with AD pathogenesis. However, the exponential growth of global research exploring GS-AD correlations poses significant challenges for knowledge integration and identification of research frontiers. While traditional systematic reviews and meta-analyses offer unique advantages in evidence synthesis, bibliometric analysis employing quantitative indicators such as citation networks and keyword co-occurrence enables more systematic revelation of domain knowledge foundations, collaboration patterns, and evolutionary trajectories (Chen et al., 2022). This large-scale scientific mapping technology not only provides researchers with panoramic domain insights but also facilitates the identification of knowledge gaps and strategic planning for innovation (Donthu et al., 2021). Notably, although existing studies have utilized bibliometric methods to analyze decade-long developmental trends in GS research (Hou et al., 2023) and conducted multidimensional examinations of AD pathological mechanism evolution (Tang et al., 2023; Zhang et al., 2024; Lin et al., 2025), there remains a critical absence of specialized bibliometric investigations focusing on the GS-AD interaction paradigm. This knowledge gap urgently requires comprehensive exploration to advance understanding of this crucial scientific question.

This study employs bibliometric methods to systematically analyze the evolution, knowledge foundations, and hotspots in GS and AD research. By constructing country/region collaboration networks, institutional synergy models, keyword clusters, and high-impact citation networks, we aim to identify core research forces, interdisciplinary trends, and potential breakthrough directions, thereby providing a theoretical framework and technical roadmap for translating basic research into clinical applications.

## Materials and methods

### Search strategy

To ensure data reliability and authority, this study utilized the Web of Science Core Collection (WOSCC) database as the data source, renowned for its high-quality peer-reviewed literature and comprehensive citation indexing system. The WOSCC comprises multiple academic indices such as the Science Citation Index Expanded (SCIE), Social Sciences Citation Index (SSCI), Index Chemicus (IC), and Current Chemical Reactions Expanded (CCRE), with advanced analytical capabilities providing critical support for citation network research. The search query was constructed as follows:

TS= (("glymphatic system" OR "glymphatic pathway" OR "Glymphatic Pathways" OR "Pathway, Glymphatic" OR "Glymphatic Clearance System" OR "glymphatic" OR "glial lymphatic" OR "Brain Perivascular Spaces" OR "Brain Perivascular Space" OR "Perivascular Space, Brain" OR "paravascular pathway") AND ("Alzheimer* disease" OR "Alzheimer* dementia" OR "AD" OR "beta-amyloid" OR "amyloid-beta" OR "Aβ" OR "tau protein" OR "neurodegeneration")).

### Eligibility criteria

The study applied specific inclusion and exclusion criteria. The inclusion criteria were: (1) peer-reviewed research articles or review articles published in English; (2) studies explicitly addressing the GS and AD or related pathological mechanisms (e.g., amyloid-β, tau protein, neurodegeneration); (3) publications from January 1, 2010, to February 7, 2025. Exclusion criteria included: (1) non-peer-reviewed materials (e.g., preprints, conference abstracts, book chapters); (2) retracted publications; (3) studies unrelated to GS or AD (e.g., focusing solely on peripheral lymphatics or unrelated neurodegenerative diseases); and (4) non-English publications to ensure consistency in data processing.

Two researchers (Liu S. C. and Zhuang L. P.) independently conducted literature screening, with disputed entries resolved through discussion with a third researcher.

### Data extraction

Extracted bibliometric parameters included: article title, publication year, author information (names, countries/regions, affiliations), citation frequency, source journal, publication type, author affiliations, keywords, and reference lists.

### Bibliometric analysis

The extracted data were imported into CiteSpace (version 6.3. R1 Advanced Edition), Microsoft Excel, RStudio (version 4.4.2), and the online bibliometric analysis platform^1^ for data processing and network visualization. CiteSpace, a pivotal analytical tool, employs visualized bibliometric methods to reveal latent insights within scientific literature. It generates scientific knowledge maps to intuitively illustrate the structure and distribution of scientific knowledge (Chen, 2004). These maps encompass multiple types, including institutional/country collaboration networks, reference co-citation clusters, burst detection maps, keyword co-occurrence networks, cluster analyses, and timeline maps, offering researchers diverse analytical perspectives. RStudio (version 4.4.2) was used with the “Bibliometrix” package to generate thematic maps and thematic evolution analyses.

In CiteSpace-generated visual knowledge networks, elements are represented through topological structures to delineate the evolutionary characteristics of the research field. Nodes, as fundamental network units, represent multiple academic entities (e.g., keywords, countries, institutions, journals), with their size being proportional to research activity or citation frequency. For further details, please refer to the relevant in-depth literature (Chen, 2006). Connecting lines between nodes denote co-occurrence relationships, forming topological links when two countries/regions or institutions collaborate on publications. A chromatic mapping mechanism integrates temporal dimensions into the visualization: cool tones (e.g., blue) represent early-year data, while warm tones (e.g., red) indicate recent research outputs, creating a visual gradient of temporal evolution. Notably, purple ring markers around nodes highlight their centrality strength, quantified by betweenness centrality to measure a node’s hub status in knowledge dissemination pathways. Nodes with high centrality typically correspond to seminal publications bridging disciplines or “knowledge gatekeepers” in social networks, exerting significant regulatory influence on domain knowledge flow (Fu et al., 2023).

The primary steps for CiteSpace software involve the following procedures. First, create a new CiteSpace project and import the complete search records obtained from the aforementioned retrieval process. Next, configure the relevant parameters within the project, including setting the time slicing to one-year intervals, performing individual annual analyses followed by merging the results, and selecting node types for analysis such as authors, keywords, journals, categories, and references. Detailed parameter configurations are provided in Supplementary Table 1. Parameters used in each analysis are specified in the upper left corner of the corresponding figure.

For reference co-citation clustering and keyword clustering analyses, the log-likelihood ratio (LLR) algorithm was applied to keywords to ensure accuracy and reliability. Generated maps display modularity (Q-value) and mean silhouette (S-value) in the upper-left corner, providing critical metrics for assessing clustering quality. Q-values range [0,1], where Q > 0.3 indicates significant clustering structure, >0.5 denotes reasonable clustering, and >0.7 reflects highly convincing clustering. For keyword and reference burst detection, γ was set to 1.0 with a minimum duration of 1 year. Keyword co-occurrence maps, cluster maps, timeline maps, and burst maps—based on keyword co-occurrence relationships in cited literature—provide comprehensive insights into keyword frequency, centrality, cluster structures, temporal spans, and thematic evolution. Timeline maps transform co-occurrence networks into chronological formats with annual legend labels, enabling intuitive observation of research trends over time (Cortese et al., 2022).

## Results

### Annual publication trends

**Figure 1:**
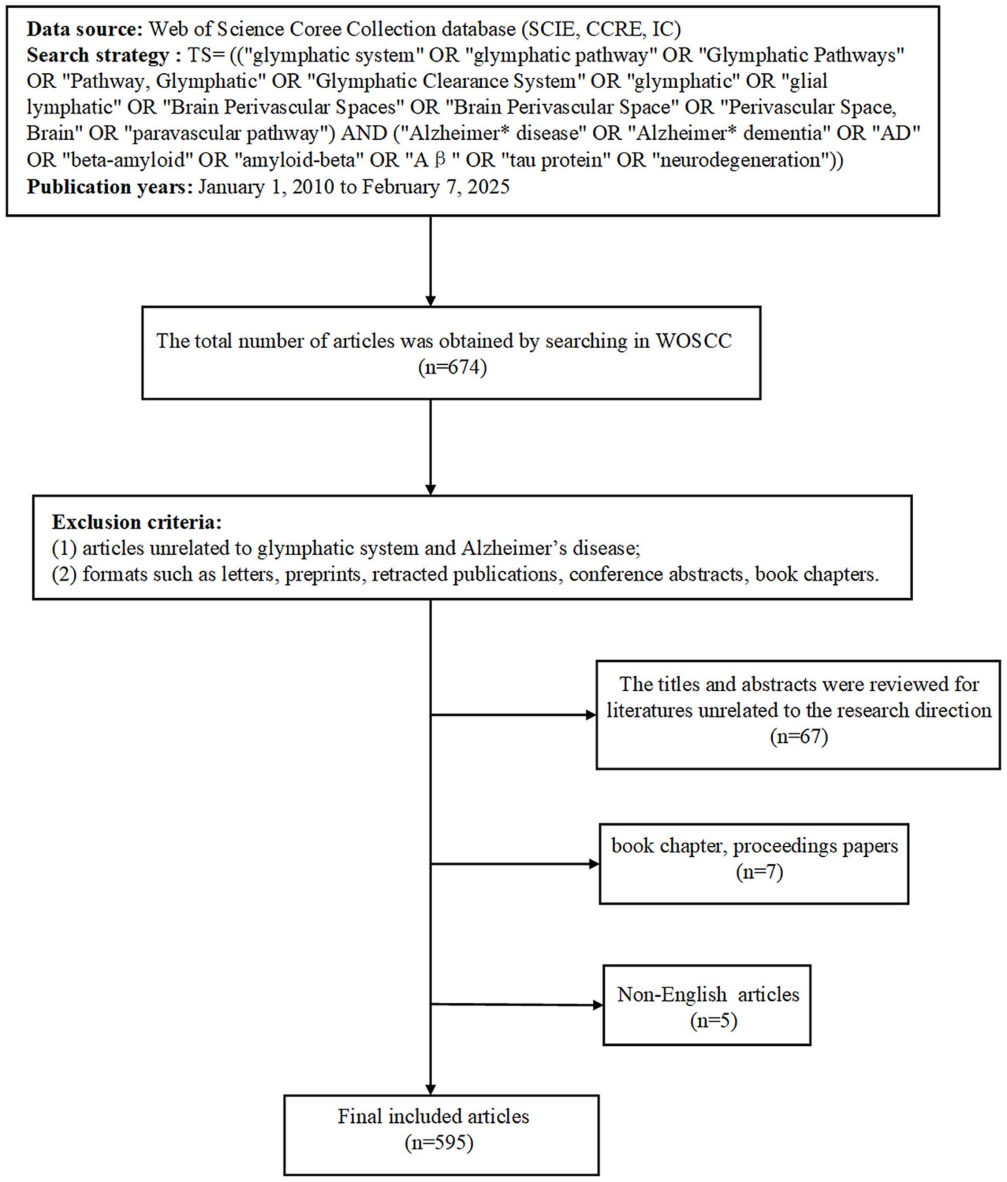
Flowchart of study identification and selection based on Web of Science Core Collection [SCIE: Science Citation Index Expanded, CCRE: Current Chemical Reactions Expanded, IC: Index Chemicus, WOSCC: Web of Science Core Collection].

**Figure 2:**
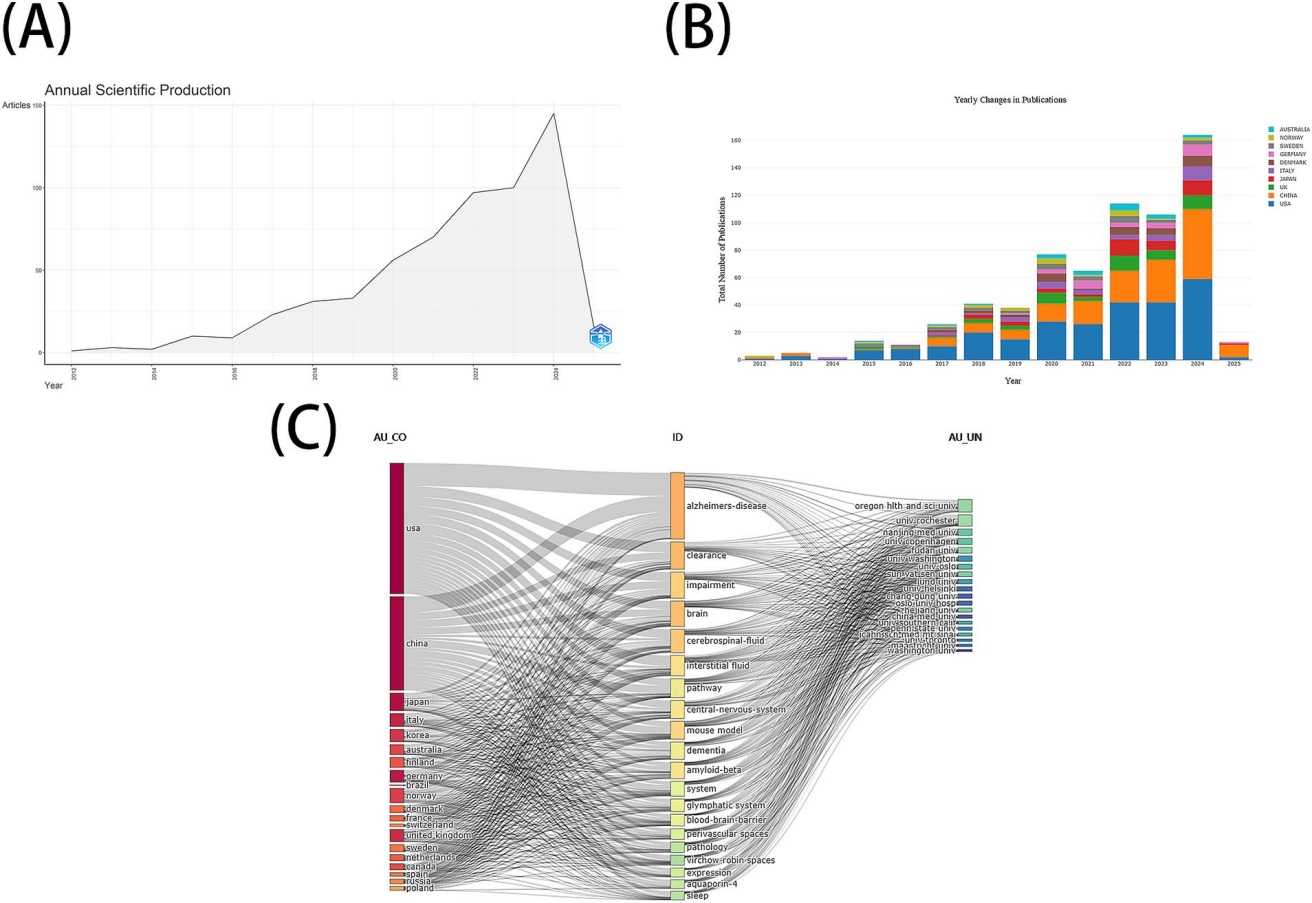
**(A)** Annual Scientific Production **(B)** The annual number of publications in major countries **(C)** RStudio—Three-fields plot left-countries, middle-keywords plus from the data records, right-authors affiliations.

The bibliographic scope of our investigation was inherently limited by the scope of our institutional database subscriptions, restricting our analysis exclusively to three curated indices within the WOSCC: SCIE, CCRE, and IC. To ensure methodological rigor, we implemented a two-stage screening protocol comprising an initial title/abstract evaluation followed by a rigorous full-text assessment. This systematic approach enabled progressive refinement of the original dataset, ultimately yielding 595 publications that met all predefined inclusion criteria. The complete selection workflow, including exclusion rationales at each filtration stage, is visually summarized in Figure 1. As shown in Figures 2A,B, research on the GS in AD has exhibited exponential growth since the seminal 2012 study by Iliff et al. (2012). Notably, publication output surged exponentially between 2015 and 2024, with 467 articles (78.49% of the total) published in the last 5 years (2020–2024). Annual output exceeded 100 articles in both 2023 and 2024, underscoring the field’s prominence in neuroscience research. Three-dimensional network analysis (country-institution-topic) (Figure 2C) revealed that the United States dominates collaborative networks, with its institutions primarily focusing on “perivascular spaces and AD pathology.” China, Japan, and Italy made significant contributions to subfields such as “cerebrospinal fluid pathway dysfunction” and “cognitive impairment.”

### Authors and co-cited authors

**Figure 3:**
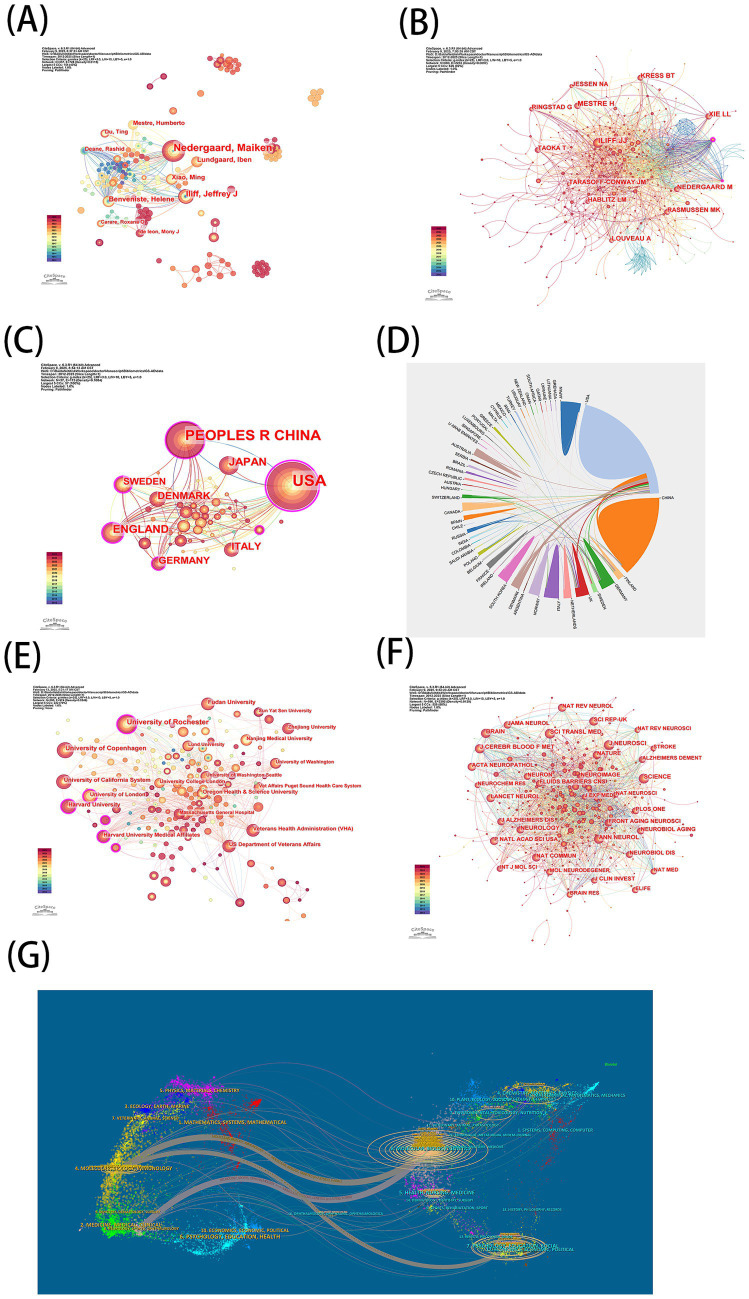
**(A)** Map of author related to glymphatic system and Alzheimer’s disease. **(B)** Map of cited author. **(C)** Map of countries **(D)** Co-operation between countries/regions. **(E)** Map of institutions **(F)** Cited journal maps. **(G)** The dual-map overlay of journals.

A total of 357 authors formed 728 collaborative links (Figure 3A). High-yield authors (≥6 publications) are listed in Table 1: Prof. Nedergaard Maiken (40 articles) from the University of Rochester Medical Center leads the field, with her team maintaining sustained influence in glymphatic mechanism research, followed by Iliff Jeffrey J, Benveniste Helene, and others. Co-cited authors—defined as two authors being jointly cited in a third author’s work—were analyzed to reveal research intensity and hot topics. The co-cited author network comprised 680 nodes and 2,232 links (Figure 3B). The top five most cited authors were: Iliff Jeffrey J (440 citations), the pioneer of glymphatic theory; Xie LL (286); Mestre H (261); Kress BT (243); and Louveau A (199). Iliff Jeffrey J’s citation dominance highlights the enduring impact of foundational studies (Table 2).

**Table 1:** The top 10 authors with the most publications.

Rank	Author	Country	Institution	Publication
1	Nedergaard, Maiken	USA	University of Rochester	40
2	Iliff, Jeffrey J	USA	University of Washington	18
3	Benveniste, Helene	USA	Yale University	11
4	Lundgaard, Iben	Sweden	Lund University	10
5	Xiao, Ming	Peoples R China	Nanjing Medical University	9
6	Chopp, Michael	USA	Henry Ford Health System	8
7	Mestre, Humberto	USA	University of Rochester	7
8	Jiang, Quan	USA	Oakland University	6
	Deane, Rashid	USA	University of Rochester	6
	Du, Ting	USA	University of Rochester	6
9	de leon, Mony J	USA	Weill Cornell Medicine	5
	Carare, Roxana O	UK	University of Southampton	5
	Arighi, Andrea	Italy	Ospedale Maggiore Policlinico	5
10	Taoka, Toshiaki	Japan	Nagoya University Graduate School of Medicine	4
	Tannenbaum, Allen	USA	Stony Brook University	4
	Plog, Benjamin A	USA	Washington University in St Louis	4
	Law, Meng	Australia	Monash University	4
	Kawoos, Usmah	USA	Naval Medical Research Center	4
	Elder, Gregory A	USA	Icahn School of Medicine at Mount Sinai	4
	Boespflug, Erin L	USA	Oregon Health & Science University	4
	Backes, Walter H	Netherlands	Maastricht University	4

**Table 2:** The top 10 authors with the most citation accounts.

Rank	Author	Country	Institution	Frequency
1	Iliff, Jeffrey J	USA	University of Washington	440
2	Xie, Lulu	USA	University of Rochester	286
3	Mestre, Humberto	USA	University of Rochester	261
4	Kress, Benjamin T	USA	University of Rochester	243
5	Louveau, Antoine	USA	Cleveland Clinic College of Medicine	199
6	Nedergaard, Maiken	USA	University of Rochester	194
7	Jessen, Nadia Aalling	USA	University of Rochester	172
8	Ringstad, Geir	Norway	University of Oslo	166
9	Rasmussen, Martin Kaag	Denmark	University of Copenhagen	163
10	Tarasoff-Conway, Jenna M	USA	New York University	161

### Country and institutional distributions

The country distribution map (57 nodes, 173 links) represents researchers from 57 countries (Figure 3C). Top countries by output and centrality are listed in Table 3. The United States, with 264 articles (44.37% of total output), leads significantly in both output and centrality (0.92), affirming its academic leadership. China ranked second in output (164 articles) but fifth in centrality, indicating abundant productivity but limited global influence. Sweden, despite lower output, ranked third in centrality (0.21), followed by the UK (0.39) and India (0.16). Network connections suggest that publications from the UK, Sweden, and Germany have acted as critical bridges across regions and periods, while other countries remain relatively isolated, necessitating enhanced international collaboration (Figure 3D).

**Table 3:** The top 10 countries with the most publications and centrality.

Rank	Publications	Countries	Rank	Centrality	Countries
1	264	USA	1	0.92	USA
2	164	Peoples R China	2	0.39	England
3	46	England	3	0.21	Sweden
4	43	Japan	4	0.16	India
5	33	Italy	5	0.14	Peoples R China
6	32	Denmark	6	0.13	Germany
7	27	Germany	7	0.09	Italy
8	24	Sweden		0.09	Australia
9	19	Australia	8	0.07	Turkey
	19	Norway	9	0.06	Netherlands
	19	Canada	10	0.04	Canada
10	17	South Korea			
	17	Netherlands			

The institutional network (280 nodes, 589 links) reflects contributions from 280 institutions (Figure 3E). The University of Rochester (45 articles) and the University of Copenhagen (31) ranked first and second in output (Table 4). Prof. Nedergaard Maiken, a renowned figure in glymphatic research, holds leadership roles at both institutions. The University of Rochester also achieved the highest centrality (0.19), followed by the University of London (0.15) and Harvard University Medical Affiliates (0.13), signifying their pivotal roles in global collaborations. Notably, Chinese institutions like Sun Yat-sen University, Fudan University, and Zhejiang University, despite high output, lack close partnerships with these leading hubs.

**Table 4:** The top 10 institutions with the most publications and centrality.

Rank	Publications	Institutions	Rank	Centrality	Institutions
1	45	University of	1	0.19	University of Rochester
2	31	Rochester	2	0.15	University of London
3	22	University of	3	0.13	Harvard University Medical Af
4	19	Copenhagen	4	0.1	filiates
	19	University of		0.1	Harvard University
5	18	California System Harvard University Fudan University University of London		0.1	Johns Hopkins University New York University
6	17	US Department of Veterans Affairs	5	0.09	Nagoya University
	17	Veterans Health Administration	6	0.08	Oregon Health & Science University
7	16	Oregon Health & Science University		0.08	University College London
8	14	Nanjing Medical University		0.08	Huazhong University of Science & Technology
	14	Zhejiang University		0.08	Sechenov First Moscow State Medical University
9	13	University College London	7	0.07	University of Copenhagen
10	12	University of Washington		0.07	US Department of Veterans Affairs
	12	Sun Yat Sen University		0.07	Centre National de la Recherche Scientifique
			8	0.06	Sun Yat Sen University
				0.06	Lund University
				0.06	University of Toronto
			9	0.05	University of California System
				0.05	Massachusetts General Hospital
				0.05	University of Texas System
			10	0.04	Veterans Health Administration

### Journal and cited journal distributions

As shown in Table 5, the top 10 journals in glymphatic-AD research published 194 articles (32.6% of total), with an average impact factor (IF) of 5.3. *Brain* had the highest IF (11.9). Journal citation network analysis (596 nodes, 2,292 links, Figure 3F) identified core journals by citation frequency and betweenness centrality (Table 6). *Science Translational Medicine*, home to Iliff Jeffrey J’s foundational study (Iliff et al., 2012), led with 470 citations, followed by Journal of *Neuroscience* (445), *Science* (440), *Brain* (399), and *Annals of Neurology* (394). Dual-map overlay analysis (Figure 3G) revealed knowledge flow patterns: citing journals clustered in “Neurology/Immunology” and “Clinical Medicine,” while cited literature originated primarily from “Molecular/Biology/Genetics,” reflecting a shift from basic to clinical research.

**Table 5:** The top 10 journals with the most publications.

Rank	Publications	Journal	IF (Quartile in category)
1	24	*Frontiers in Aging Neuroscience*	4.1 (*Q2*)
2	22	*International Journal of Molecular Sciences*	4.9 (*Q1*)
3	20	*Journal of Alzheimer’s Disease*	3.4 (*Q2*)
4	17	*Frontiers in Neuroscience*	3.2 (*Q2*)
5	15	*Journal of Cerebral Blood Flow and Metabolism*	4.9 (*Q1*)
6	15	*Frontiers in Neurology*	2.7 (*Q2*)
7	13	*Fluids and Barriers of the CNS*	5.9 (*Q1*)
8	12	*Scientific Reports*	3.8 (*Q1*)
9	11	*Neurobiology of Disease*	5.1 (*Q1*)
	10	*Aging and Disease*	7.0 (*Q1*)
10	7	*Alzheimers & Dementia*	4.9 (*Q1*)
	7	*Alzheimers Research & Therapy*	8.0 (*Q1*)
	7	*Brain*	11.9 (*Q1*)
	7	*Journal Of Magnetic Resonance Imaging*	3.3 (*Q1*)
	7	*Neural Regeneration Research*	5.9 (*Q1*)

**Table 6:** The top 10 journals by citation frequency and top 5 journals by network centrality.

Rank	Cited journal	Frequency	Rank	Cited journal	Centrality
1	*Sci Transl Med*	470	1	*Adv Neurol*	0.09
2	*J Neurosci*	445	2	*Acta Neurol Scand*	0.06
3	*Science*	440		*Aging Cell*	0.06
4	*Brain*	399	3	*Am J Neuroradiol*	0.05
5	*Ann Neurol*	394		*Curr Alzheimer Res*	0.05
6	*J Cerebr Blood F Met*	372	4	*Behav Brain Res*	0.04
7	*Neurology*	342		*Brain Res Rev*	0.04
8	*P Natl Acad Sci Usa*	341		*Neurotherapeutics*	0.04
9	*J Alzheimers Dis*	336		*Cns Neurol Disord-Dr*	0.04
10	*Sci Rep-UK*	333		*Am J Physiol-Reg I*	0.04
				*Acta Neurochir Suppl*	0.04
			5	*Brain Pathol*	0.03
				*Alzheimers Res Ther*	0.03
				*J Neurochem*	0.03
				*Curr Alzheimer Res*	0.03
				*Neurotherapeutics*	0.03

### Cited reference analysis

**Figure 4:**
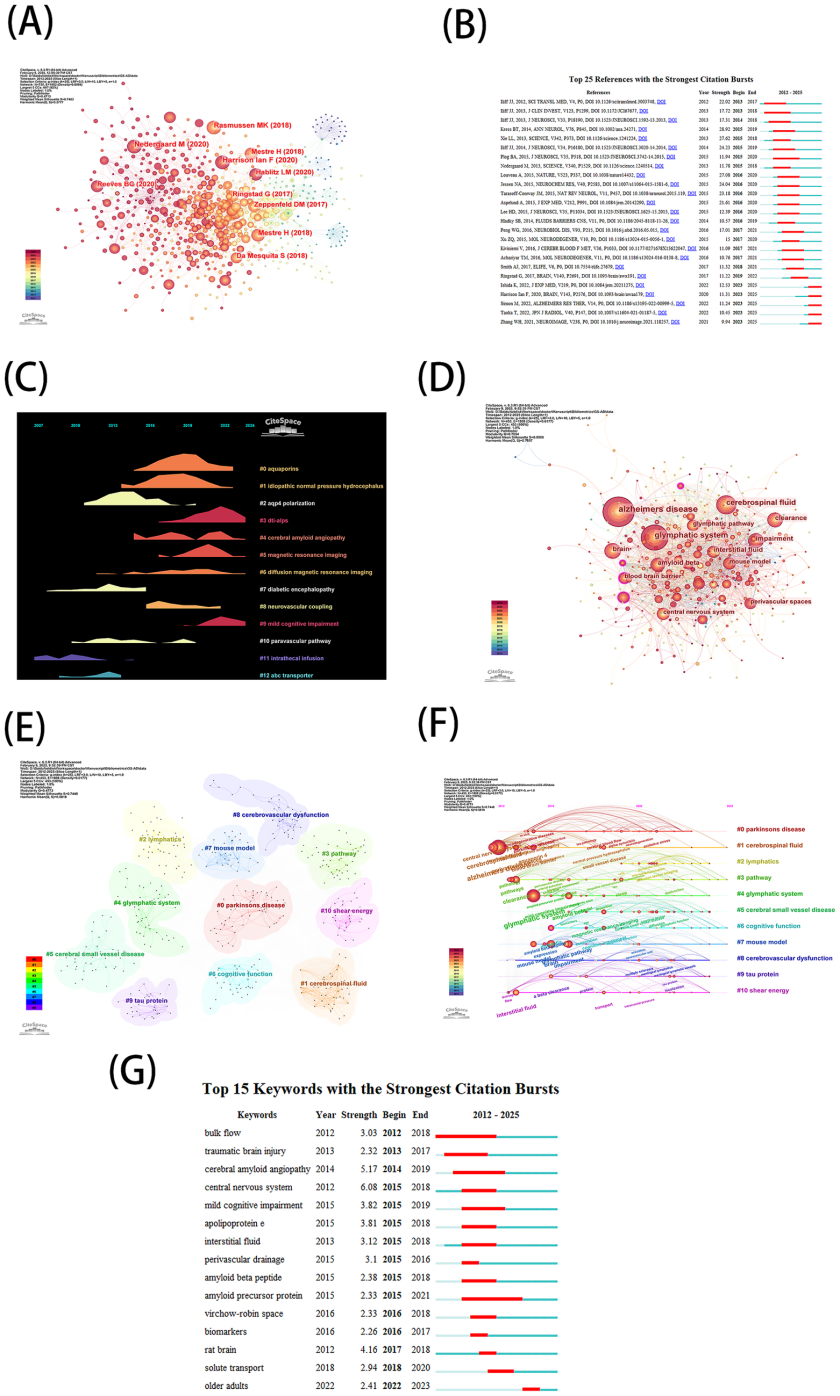
**(A)** Map of cited references **(B)** The top 25 references with the strongest citation bursts. The blue bars indicate that the reference has been published; the red bars indicate citation burstness. **(C)** The visualization map of the timeline viewer. **(D)** Map of keywords occurrence **(E)** The clustering of keywords. **(F)** Time dynamic evolution of keywords **(G)** The top 15 keywords with the strongest citation bursts. The blue bars indicate that the reference has been published; the red bars indicate citation burstness.

The co-citation network (755 nodes, 1,952 links; Figure 4A) highlights the field’s knowledge foundation. Table 7 lists the top 10 most cited references, including: “Impaired glymphatic function and clearance of tau in an Alzheimer’s disease model”(Harrison et al., 2020), “Glymphatic failure as a final common pathway to dementia”(Nedergaard and Goldman, 2020), and “The glymphatic pathway in neurological disorders”(Rasmussen et al., 2018), all exceeding 100 citations. Burst detection identified 25 references with accelerated citation rates (Figure 4B). Bursts began 1–2 years post-publication, first appearing in 2013 and peaking between 2015 and 2017, with five high-intensity bursts since 2023. Timeline clustering (Figure 4C) revealed temporal spans of research themes. While “aquaporins” and “idiopathic normal pressure hydrocephalus” dominated early discussions, emerging topics like “cerebral amyloid angiopathy,” “DTI-ALPS,” and “mild cognitive impairment” have gained recent traction.

**Table 7:** The top 10 cited references with the most citation counts references.

Rank	Frequency	Article title	Authors & Year	Main Findings
1	113	Impaired glymphatic function and clearance of tau in an Alzheimer’s disease model.	Harrison et al. (2020)	AQP4 dysfunction reduces tau clearance, exacerbating Alzheimer’s pathology.
2	110	Glymphatic failure as a final common pathway to dementia.	Nedergaard and Goldman (2020)	Glymphatic dysfunction links aging, sleep loss, and neurodegenerative protein accumulation.
3	101	The glymphatic pathway in neurological disorders.	Rasmussen et al. (2018)	Glymphatic impairment observed in Alzheimer’s, TBI, and other neurological diseases.
4	92	Aquaporin-4-dependent glymphatic solute transport in the rodent brain.	Mestre et al. (2018a)	AQP4 is critical for glymphatic transport; anesthesia and age affect results.
5	90	Circadian control of brain glymphatic and lymphatic fluid flow.	Hablitz et al. (2020)	Glymphatic influx peaks during rest phase; AQP4 polarization drives circadian CSF dynamics.
6	88	Glymphatic System Impairment in Alzheimer’s Disease and Idiopathic Normal Pressure Hydrocephalus.	Reeves et al. (2020)	Both Alzheimer’s and iNPH exhibit AQP4 misloca lization and reduced glymphatic clearance.
	88	Flow of cerebrospinal fluid is driven by arterial pulsations and is reduced in hypertension.	Mestre et al. (2018b)	Arterial pulsations drive CSF flow; hypertension reduces flow efficiency.
7	82	Functional aspects of meningeal lymphatics in ageing and Alzheimer’s disease.	Da Mesquita et al. (2018)	Meningeal lymphatics drain CNS waste; dysfunction worsens Alzheimer’s pathology and cognitive decline.
8	78	Glymphatic MRI in idiopathic normal pressure hydrocephalus.	Ringstad et al. (2017)	iNPH patients show delayed CSF clearance and reduced glymphatic function via MRI.
	78	Association of Perivascular Localization of Aquaporin-4 With Cognition and Alzheimer Disease in Aging Brains.	Zeppenfeld et al., 2017	Loss of perivascular AQP4 correlates with amyloid burden and Alzheimer’s progression.
9	77	Increased glymphatic influx is correlated with high EEG delta power and low heart rate in mice under anesthesia.	Hablitz et al. (2019)	Glymphatic influx is highest under K/X anesthesia in mice, positively correlating with EEG delta power and negatively with heart rate.
10	75	The Brain’s Glymphatic System: Current Controversies.	Mestre et al. (2020)	Discusses glymphatic controversies: flow direction, AQP4 function, ICP effects, and Aβ clearance diurnality.

### Keyword analysis

**Figure 5:**
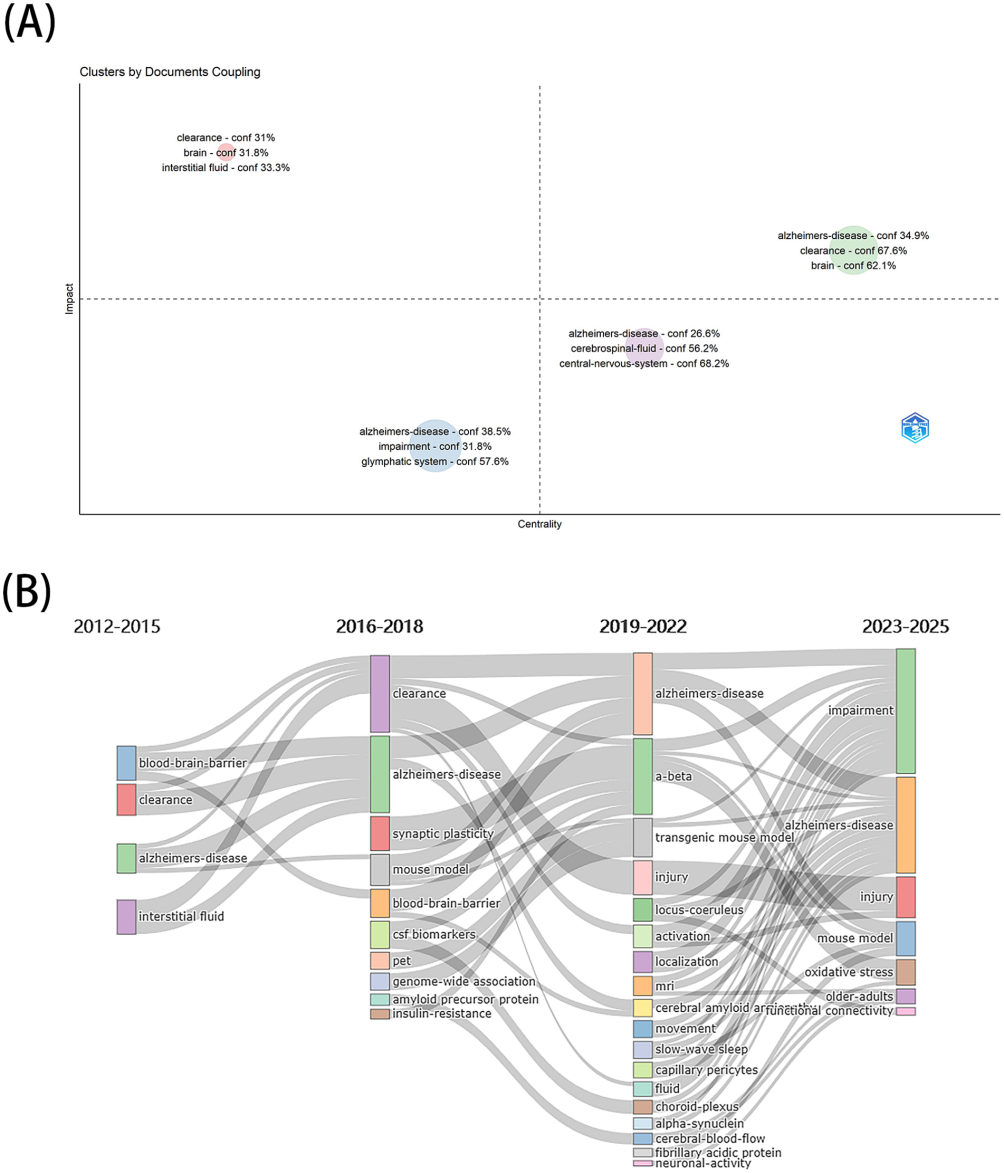
**(A)** The strategy map of identified topics clustered by keywords plus. **(B)** Thematic Evolution of glymphatic system and Alzheimer’s disease research from 2012 to 2025.

The keyword co-occurrence network (453 nodes, 1,808 links; Figure 4D) identified high-frequency and high-centrality terms (Table 8), including “Alzheimer’s disease” (399), “glymphatic system” (308), “cerebrospinal fluid,” “interstitial fluid,” “Aβ clearance,” “amyloid beta,” “blood–brain barrier,” “aquaporin-4,” and “cognitive function.” Cluster analysis grouped keywords into 11 thematic clusters (Figure 4E), such as #0 Parkinson’s disease, #1 cerebrospinal fluid, and #4 glymphatic system. Timeline visualization (Figure 4F) showed thematic shifts: “glymphatic system” and “tau protein” dominated from 2012 to 2020, while “lymphatics” and “cerebrospinal fluid” surged post-2020. Burst detection (Figure 4G) highlighted emerging keywords like “solute transport” and “older adults” (2020–2024), signaling current and future research directions. This study constructed a strategic coordinate map to reveal disciplinary thematic structures through clustering analysis of the top 250 high-frequency keywords (with a minimum frequency threshold of 40). By extracting three core labels for each theme, a four-quadrant analytical framework was established using centrality (X-axis) and impact (Y-axis) as coordinates (Figure 5A). Centrality characterizes a theme’s hub status within the disciplinary network, while impact reflects its developmental maturity. The first quadrant (high centrality-high impact) represents “motor themes” that embody mainstream research directions. The second quadrant (low centrality-high impact) identifies “niche themes” with independent, mature research systems. The third quadrant (low centrality-low impact) corresponds to “emerging/declining themes,” and the fourth quadrant (high centrality-low impact) highlights “basic themes” requiring deeper exploration. Thematic evolution (Figure 5B) divided the timeline into four phases (2012–2015, 2016–2018, 2019–2022, 2023–2025), illustrating a transition from mechanistic exploration to clinical translation.

**Table 8:** The top 10 keywords by frequency and top 5 keywords by network centrality.

Rank	Frequency	Keyword	Rank	Centrality	Keyword
1	399	Alzheimers disease	1	0.14	Dementia
2	308	Glymphatic system	2	0.11	Blood brain barrier
3	178	Cerebrospinal fluid		0.11	Amyloid beta peptide
4	125	Clearance		0.11	Choroid plexus
5	119	Brain	3	0.1	Mild cognitive impairment
6	100	Impairment	4	0.09	A beta clearance
7	92	Interstitial fluid	5	0.08	Aquaporin 4
8	91	Amyloid beta		0.08	Cognitive function
9	87	Mouse model			
10	85	Perivascular spaces			

## Discussion

This study, based on 595 documents from the WOSCC, systematically integrated the spatiotemporal distribution, knowledge base, and cutting-edge topics in the field of the GS and AD for the first time. The research revealed a “dual—phase development” characteristic in this field: from 2012 to 2014, it was the theoretical validation period, with an average of less than 10 publications per year; after 2015, it entered an exponential growth phase, with the number of publications in 2024 (144) being 14.4 times that in 2015 (10) (Figure 2A). The emergence of this turning point may be closely related to two milestone studies: the Nedergaard team’s first systematic elaboration of the molecular mechanisms of the glymphatic system (Jessen et al., 2015), and Louveau et al.’s discovery of the functional association between meningeal lymphatic vessels and the peripheral immune system (Louveau et al., 2015). The current research growth rate (15 publications in January 2025) indicates that this field will continue to lead the forefront of neuroscience.

In terms of national and research institution distribution, the United States holds an academic dominant position in this research field, with Professor Nedergaard M.’s University of Rochester making significant contributions and establishing extensive and close cooperative relationships with developed European countries such as Denmark and the United Kingdom. China, as the largest developing country with the largest base of AD patients in the world, has made significant progress in research funding and document output in recent years, relying on research institutions such as Fudan University, Sun Yat—sen University, Zhejiang University, and Nanjing Medical University. However, its hub status in the international cooperation network is still significantly lower than that of European and American developed countries. This indicates that Chinese scholars need to focus on improving the academic impact of their achievements while ensuring the expansion of research scale, and break through the current quality bottleneck by optimizing the rigor of research design and clinical transformation value. To improve the current unbalanced global scientific research resource allocation, it is recommended to build an international knowledge—sharing platform led by the World Health Organization, promote the efficient transformation of basic research results into clinical practice through cooperation mechanisms such as establishing cross-national research alliances, standardized data collection protocols, and multi-center clinical trials.

Among the related research fields, the author with the highest number of publications is Professor Nedergaard M. Her team, through systematic research, has profoundly revealed the GS as a key core mechanism for waste clearance in the brain. They pioneeringly confirmed that the system can efficiently clear metabolic products such as Aβ protein through the CSF-ISF exchange pathway during sleep, and further clarified its close association with neurodegenerative diseases such as AD. In addition, the team’s research also found that the AQP4 plays a crucial role in the function of the GS, and revealed the destructive mechanisms of external and physiological factors such as air pollution and aging on the system, expanding the research perspective to the fields of eye diseases and brain injuries. Their related achievements provide valuable theoretical support and practical foundation for the development of neuroscience and disease treatment (Ren et al., 2013; Xie et al., 2013; Nedergaard and Goldman, 2020). It is worth mentioning that XIE LL, who ranks second in the co-citation author ranking, is under his guidance. The following author is Iliff, Jeffrey J., who, as the discoverer of the GS, also ranks first in the co-citation authors, fully demonstrating his significant position in this field. Iliff, Jeffrey J. first systematically revealed the mechanism by which CSF clears brain metabolic waste (such as Aβ) through the vascular—perivascular pathway (i.e., the GS). His subsequent research further confirmed the close association between glymphatic dysfunction and the pathology of diseases such as AD and traumatic brain injury, providing a solid theoretical basis for the formulation of treatment and prevention strategies for neurodegenerative diseases (Iliff et al., 2012, 2014; Zeppenfeld et al., 2017; Mestre et al., 2018a). The third—ranked author is Benveniste, Helene, whose main contribution lies in the detailed elaboration of the flow mechanism of cerebrospinal fluid through the PVS in the brain, which is crucial for the clearance of brain metabolic waste. Her research achievements not only deepened the academic community’s understanding of the brain’s self-cleaning process but also opened up a new perspective for exploring the pathogenesis of AD and potential treatment methods (Benveniste et al., 2017).

Through co-citation visualization analysis, it was found that the article with the highest number of co-citations was “Impaired glymphatic function and clearance of tau in an Alzheimer’s disease model” published by Harrison Ian F. et al. in 2020. The study pioneeringly revealed the direct association between GS dysfunction and tau protein clearance disorders in a tau-lesion mouse model, clearly clarified the key regulatory role of AQP4 polarization in the glymphatic-mediated tau clearance process, and found a correlation between brain-region-specific glymphatic function differences and tau pathological distribution, providing new molecular targets and potential treatment directions for AD pathogenesis research (Benveniste et al., 2017). The second-ranked was the study published by Nedergaard M. et al. in “*Science*,” which deeply revealed the close internal connections between the GS, sleep, aging, and neurodegenerative diseases, proposing that GS failure is the core pathway for the development of various dementias. At the same time, the study integrated the synergistic damaging effects of factors such as cardiovascular health, abnormal AQP4 localization, and decreased sleep quality on glymphatic function, and pointed out that improving sleep or targeting enhanced glymphatic clearance ability may become a new strategy for treating neurodegenerative diseases (Nedergaard and Goldman, 2020). The remaining documents emphasized the important role of the GS in various nervous system diseases and its huge potential as a treatment target from different dimensions. These documents covered the roles of the GS in CSF flow, clearance of extracellular substances, and circadian rhythm regulation (Zeppenfeld et al., 2017; Da Mesquita et al., 2018; Mestre et al., 2018a, 2018b; Hablitz et al., 2020); revealed its functional disorders in diseases such as AD and idiopathic normal-pressure hydrocephalus, leading to the accumulation of toxic proteins and neurodegenerative changes (Reeves et al., 2020); In addition, with the development of glymphatic magnetic resonance imaging (MRI) technology, such as DTI-ALPS technology, non-invasive assessment of GS function has become possible, which is helpful for early disease diagnosis and progression monitoring, and has opened up new avenues for therapeutic interventions aimed at enhancing glymphatic function to improve cognitive outcomes and delay disease progression (Ringstad et al., 2017). In short, these research achievements have paved the way for scholars new to the field to carry out further research, providing a valuable research foundation and direction guidance.

The journal distribution characteristics in the field of the GS and AD indicate that research results are concentrated in high-impact journal clusters. The top three journals, *Frontiers in Aging Neuroscience*, *International Journal of Molecular Sciences*, and *Journal of Alzheimer’s Disease*, have made significant contributions to the total number of publications, and their academic credibility and field-focused nature provide scholars with a precise platform for publishing their achievements. It is worth noting that all the top 10 journals are ranked *Q2* or above in the Journal Citation Reports division (with *Q1* journals accounting for 73.3%), which confirms that the theoretical depth and clinical transformation value of research in this field have been highly recognized by international peers. The analysis of the citation journal network further revealed that the academic influence of GS research has a cross-disciplinary diffusion characteristic. The frequent citation of high-impact journals such as “*Science Translational Medicine*” (cited 470 times), “*Journal of Neuroscience*” (445 times), and “*Science*” (440 times) not only reflects the core position of basic mechanism research (such as AQP4 polarization regulation and CSF dynamics) but also reflects the deep integration trend between GS theory and clinical research on neurodegenerative diseases. This “basic-clinical” two-way citation pattern highlights the unique value of the GS as an interdisciplinary hub, and its research achievements, through the dissemination of top-tier journals, continue to promote the interdisciplinary integration of neuroscience, imaging medicine, and translational medicine.

Through bibliometric analysis, this study systematically elaborated the international development trend of the research field of the GS and AD, and refined three core research directions: First, the anatomical basis and fluid dynamics characteristics of the GS (involving cerebrospinal fluid CSF, interstitial fluid ISF, lymphatic vessels, perivascular drainage pathways, etc.); Second, the physiological functions of the GS and its regulatory mechanisms for cognitive impairment (including metabolic clearance, Aβ, tau protein, AQP4, etc.); Third, the translational medical value of the GS in AD diagnosis and treatment (such as DTI-ALPS imaging biomarkers, biomarker development, and intrathecal drug delivery and other treatment strategies).

In terms of anatomical mechanism research, Iliff et al. (2012) first confirmed the dynamic exchange of CSF and ISF through PVS, and the system was named the GS due to its functional similarity to the peripheral lymphatic system (Iliff et al., 2012). Subsequently, the Ringstad team obtained the first human clinical evidence of GS function through tracer imaging technology (Ringstad et al., 2018). It is worth noting that the normal operation of the GS is highly dependent on the circulatory dynamics of CSF (Chong et al., 2022). Yamamoto et al. (2024) used magnetic resonance imaging technology to directly observe the transport path of CSF tracers in the PVS of the living human brain for the first time, providing important visual evidence for the anatomical structure of the GS (Yamamoto et al., 2024). Traditional theory holds that CSF is mainly drained into the venous sinuses through arachnoid granulations (AG), but recent research has revealed a more complex multi-pathway drainage mechanism: including the dural lymphatic pathway (drained to the deep cervical lymph nodes after interacting with the meningeal lymphatic vessels through the arachnoid cuff exit [ACE])(Louveau et al., 2015) and the perineural pathway (flowing out along the PVS of cranial and spinal nerves)(Proulx, 2021; Ligocki et al., 2024). It is particularly important to note that the ACE structure has a unique liquid-molecule bidirectional exchange regulatory function, serving both as a transport channel and a reverse diffusion barrier. In addition, the anatomical-functional synergy mechanism between the dural lymphatic vessels and the venous system plays a key role in drainage regulation. Future research needs to focus on solving the following scientific questions: quantifying the dynamic contributions and compensatory relationships of different drainage pathways (dural lymphatic, perineural, and residual AG pathways); clarifying the molecular regulatory mechanisms and pathological significance of special structures such as ACE; elucidating the spatiotemporal specificity of meningeal lymphatic drainage in central-peripheral immune interaction; and establishing a causal association model between GS dysfunction and neurodegenerative diseases. It is worth noting that the deep cervical lymphatic-venous drainage surgery developed based on the theory of dural lymphatic flow has shown significant therapeutic effects in AD clinical treatment. This surgical procedure, which reconstructs the lymphatic drainage pathway through microsurgical technology, has been widely promoted and applied in clinical practice (Xie et al., 2023).

At the pathological mechanism level, AD, as a typical representative of age-related neurodegenerative diseases, is characterized by the abnormal aggregation of Aβ and the formation of neurofibrillary tangles by over-phosphorylated tau protein (Selkoe, 2003). The GS plays a key role in the clearance of metabolic products, nutrient delivery, volume regulation, and immune homeostasis maintenance through its unique material transport function (Rasmussen et al., 2022). Specifically, the system can effectively clear neurotoxic substances such as tau protein, Aβ, and α-synuclein. When the clearance capacity of the GS is impaired, it will lead to the abnormal accumulation of Aβ, thereby accelerating the pathological process of AD (Pedersen et al., 2023). Da Mesquita et al. (2018) found the specific accumulation of Aβ in the cervical lymph nodes, providing direct evidence for the Aβ clearance hypothesis of the GS (Nauen and Troncoso, 2021). AQP4, as a key molecule regulating CSF-ISF exchange, has a decisive impact on maintaining the clearance efficiency of the GS with its polar distribution characteristic (about 50% expressed on the perivascular end-feet of astrocytes)(Nielsen et al., 1997; Simon et al., 2022). Studies have shown that AQP4 accelerates the clearance of pathogenic proteins such as Aβ by promoting the flow of extracellular fluid, and its dysfunction may lead to the pathological progression of AD (Iliff et al., 2012). It is worth noting that the role of the GS in Parkinson’s disease (PD) is also increasingly attracting attention. PD shares similar protein misfolding pathological characteristics with AD, and its pathogenic mechanism involves the abnormal deposition of α-synuclein forming Lewy bodies (Luk et al., 2012). The latest research shows that the GS is also involved in the clearance process of α-synuclein (Luk et al., 2012), which suggests that cross-disease joint research on AD and PD may produce synergistic innovative effects.

The function of the GS is dynamically affected by multi-dimensional regulatory mechanisms, mainly involving physiological rhythms, vascular hemodynamics, and aging factors. In terms of physiological rhythms, slow-wave activity during non-rapid eye movement sleep can significantly enhance the clearance efficiency of the GS, and the increased CSF flow during sleep promotes the clearance of metabolic products such as Aβ (Xie et al., 2013). The polar distribution of AQP4 is regulated by the circadian rhythm, and the reduction of core body temperature can enhance the function of the GS (Hablitz et al., 2020; Yao et al., 2023). The aging process is accompanied by weakened vascular pulsation, abnormal polar distribution of AQP4, and structural changes in meningeal lymphatic vessels, leading to a significant decrease in CSF-ISF exchange efficiency (Selkoe, 2003; Kress et al., 2014; Ahn et al., 2019). At the vascular hemodynamics level, the arterial pulsation generated by cardiac contraction drives CSF to flow along the PVS through the “pump effect” formed by vascular wall displacement (Mestre et al., 2018b); while respiratory movement regulates CSF flow speed through changes in intrathoracic pressure (Wilson, 2016). In-depth parsing of these regulatory mechanisms provides an important theoretical basis for the development of AD intervention strategies.

In terms of translational medical applications, the GS has dual potential in the diagnosis and treatment of AD. Imaging studies have confirmed that diffusion tensor imaging-based PVS analysis (DTI-ALPS) can effectively assess the function of the GS in AD patients. Clinical data show that the ALPS index of AD patients is significantly lower than that of healthy controls, and this index is significantly correlated with key pathological indicators such as the degree of cognitive decline, Aβ/tau deposition levels, and gray matter integrity, indicating its potential value as a diagnostic biomarker for AD (Taoka et al., 2017; Steward et al., 2021; Ota et al., 2022; Chang et al., 2023). It is crucial to emphasize that DTI-ALPS does not constitute a direct measurement of the GS. This technical parameter fundamentally reflects the diffusion properties of water molecules in the interstitial space along the direction of the PVS (Taoka et al., 2017). Existing studies suggest that these diffusion characteristics may correlate with GS activity; however, the precise relationship between these diffusion properties and GS functionality remains a subject of ongoing investigation and academic debate in contemporary neuroscience (Ringstad, 2024). Notably, while DTI-ALPS offers a novel non-invasive approach for studying glymphatic function, significant advancements have also been achieved in other neuroimaging-based GS biomarker research. Major developments include deep learning-based PVS quantification segmentation (Sepehrband et al., 2019), multiphysics-coupled modeling of CSF distribution mapping (Zhou et al., 2024), tracer-enhanced PET-CSF clearance kinetic analysis (Schubert et al., 2019; Li et al., 2022), dynamic diffusion-weighted imaging (DWI) based CSF flow visualization (Wen et al., 2022), and diffusion-prepared arterial spin labeling techniques for blood–brain barrier permeability assessment (Shao et al., 2023). The synergistic application of these multimodal imaging technologies not only refines the evaluation framework for neurometabolic waste clearance systems but also provides new research paradigms for elucidating their mechanistic roles in cerebral homeostasis maintenance and neurodegenerative disease pathogenesis. Although the GS may serve as a pathway for pathological protein spread, its characteristic as a natural delivery pathway also provides new ideas for targeted therapy. Studies have shown that adeno-associated virus vectors (Murlidharan et al., 2016) and nanoparticle carriers (Gu et al., 2020) can use the GS to achieve efficient delivery of therapeutic agents. In addition, non-invasive methods such as focused ultrasound, photobiomodulation therapy, and exercise intervention enhance glymphatic function through mechanisms such as enhancing vascular pulsation, regulating AQP4 polarity, or improving lymphatic drainage, opening up new avenues for AD treatment (Von Holstein-Rathlou et al., 2018; Lee et al., 2020; Baik et al., 2021).

As the core regulatory network for metabolic clearance and substance transport in the central nervous system, future research on the GS should focus on multi-dimensional mechanism parsing and clinical translation innovation, mainly covering the following cutting-edge directions: First, integrating high-resolution *in-vivo* imaging techniques with computational fluid dynamics models to systematically clarify the driving mechanisms of glymphatic flow (including the synergistic effects of multiple physical fields such as pressure gradients, osmotic pressure differences, and arterial pulsations), and revealing its dynamic regulatory patterns under physiological activities such as the sleep-wake cycle and cardiovascular function; Second, establishing a causal association model between glymphatic clearance dysfunction and neurodegenerative diseases (such as AD and PD), focusing on exploring neuroprotective strategies to enhance Aβ clearance efficiency through regulating the polar expression of AQP4 or optimizing slow-wave sleep rhythms; Third, there is an urgent need to develop advanced imaging methods based on technologies such as dynamic contrast-enhanced MRI to achieve real-time dynamic monitoring of glymphatic transport processes, and to accelerate the clinical translation of targeted intervention plans by establishing a standardized quantitative evaluation system for cerebrospinal fluid tracer clearance rates; Finally, existing clinical studies have accumulated a large number of GS function indicators related to the pathological process of AD (such as DTI-ALPS index and CSF clearance rate), so there is an urgent need to conduct multi-center evidence-based medical studies and formulate clinical practice guidelines to promote the integrated application of related biomarkers in the early diagnosis of AD, and to establish the improvement of glymphatic system function as a clinically feasible therapeutic target.

### Strengths and limitations

To our knowledge, this study represents the first knowledge-mapping exploration specifically targeting the association between the GS and AD. Utilizing multiple bibliometric analysis tools combined with co-word analysis and systematic literature review, this research elucidates annual publication trends, regional/institutional collaboration networks, co-cited literature clusters, core journal distributions, and thematic evolution patterns. However, several methodological limitations should be acknowledged. Firstly, the data collection cutoff date was February 7, 2025, and given the continuous updates to the WOSCC database—particularly the ongoing inclusion of 2025 publications—there may be delays in capturing the latest disciplinary advancements. Secondly, this study encountered two principal methodological constraints. The technical limitations inherent in CiteSpace’s data processing framework mandated exclusive utilization of WOSCC data, inherently restricting source diversity. Compounding this challenge, institutional access limitations confined our WOSCC search strategy to three primary citation indices (SCIE, CCR, IC), thereby excluding potentially relevant social science literature indexed in SSCI. This dual restriction protocol precluded the inclusion of valuable research from prominent biomedical repositories (PubMed, EMBASE) and regionally significant scientific output cataloged in SciELO. These combined limitations highlight the critical importance of developing interoperable analytical platforms capable of standardizing heterogeneous data formats. Furthermore, they underscore the necessity for establishing systematic integration frameworks for multidisciplinary databases to enable truly comprehensive bibliometric analyses that reflect global scientific discourse. Thirdly, the English-language filter may have excluded high-quality studies published in other languages (e.g., Chinese, Japanese, German), potentially limiting the global generalizability of findings. Finally, visual analyses based on CiteSpace’s built-in algorithms may exhibit clustering threshold sensitivity; although parameter optimization improved interpretability, qualitative synthesis remains essential for result validation. It is noteworthy that the knowledge framework constructed in this study aligns closely with current disciplinary advancements, and its revealed evolutionary patterns offer reliable references for future research.

## Conclusion

Through longitudinal bibliometric analysis, this study systematically delineated the evolutionary trajectory and knowledge map of GS-AD research over the past 15 years. The field exhibits dynamic expansion without reaching a plateau, indicating substantial potential for sustained innovation. The United States leads the field, with the University of Rochester serving as the pivotal institution driving breakthroughs. Current research focuses on three core scientific questions: the anatomical characterization of the glymphatic system, its physiological regulatory mechanisms, and its translational value in AD diagnosis and therapy. Looking ahead, novel neuroimaging technologies such as dynamic contrast-enhanced MRI, combined with multi-omics biomarker integration, are poised to deepen mechanistic insights. Concurrently, a collaborative framework bridging basic and clinical research is urgently needed to elucidate the spatiotemporal specificity of glymphatic dysfunction in early AD pathophysiology. By fostering cross-disciplinary integration and innovative therapeutic paradigms, the GS emerges as a promising target for precision medicine in AD, offering transformative potential for early intervention and personalized treatment strategies.

## Data Availability

The original contributions presented in the study are included in the article/Supplementary material, further inquirieas can be directed to the corresponding author.

## Footnotes

^1^ http://bibliometric.com/

## Supplementary material

The Supplementary material for this article can befound online at: https://www.frontiersin.org/articles/10.3389/fnagi.2025.1579373/full#supplementary-material
