# Supplementary material for: Research hotspots and frontiers of glymphatic system and Alzheimer’s disease: a bibliometrics analysis
Source: Front Aging Neurosci. 2025 Jul 2;17:1579373. doi: 10.3389/fnagi.2025.1579373 (PMC12263927; doi:10.3389/fnagi.2025.1579373)
Supplement: Supplementary file 1 [file Table_1.docx]

| **Supplementary Table 1** Parameter settings for CiteSpace | | | |
| --- | --- | --- | --- |
| Parameter | | Description | Impact |
| g-index(k=25) | For each temporal segment, the most cited 25% of publications were chosen | | Eliminates less influential works to emphasize pivotal studies |
| LRF=3.0 | Link Retention Factor (LRF) regulates the persistence intensity of connections over chronological intervals | | Increased values enhance temporal continuity between linked elements |
| L/N=10 | Limit each node to a maximum of 10 connections | | Reduces network intricacy to maintain analytical clarity |
| LBY=5 | Temporal analysis window restricted to the most recent five-year period | | Prioritizes contemporary research trends and emerging domains |
| E=1.0 | Inter-temporal connection weighting coefficient | | A value of 1.0 maintains equivalent weighting for historical and current linkages |

| Abbreviations | Full name |
| --- | --- |
| AD | Alzheimer's Disease |
| Aβ | Amyloid-β |
| GS | Glymphatic System |
| PVS | Perivascular Spaces |
| CSF | Cerebrospinal Fluid |
| ISF | Interstitial Fluid |
| AQP4 | Aquaporin-4 |
| DTI-ALPS | Diffusion Tensor Image Analysis Along the Perivascular Space |
| WOSCC | Web Of Science Core Collection |
| LLR | Log-Likelihood Ratio |
| IF | Impact Factor |
| AG | Arachnoid Granulations |
| ACE | Arachnoid Cuff Exit |
| PD | Parkinson's Disease |
| MRI | Magnetic Resonance Imaging |
| TBI | Traumatic Brain Injury |
| CNS | Central Nervous System |
| iNPH | idiopathic Normal Pressure Hydrocephalus |

**Supplementary materials**
